# *PGMweb*: an online tool for visualizing the X-ray beam path through plane grating monochromators

**DOI:** 10.1107/S1600577524011603

**Published:** 2025-01-01

**Authors:** Patrick Yuheng Wang, Murilo Bazan da Silva, Matthew Hand, Hongchang Wang, Peter Chang, Victoria Beilsten-Edmands, Timur K. Kim, Tien-Lin Lee, Kawal Sawhney, Andrew C. Walters

**Affiliations:** a Diamond Light Source Harwell Science and Innovation Campus Didcot OxfordshireOX11 0DE United Kingdom; b EaSTCHEM School of Chemistry University of Edinburgh EdinburghEH9 3FJ United Kingdom; Australian Synchrotron, Australia

**Keywords:** monochromator, plane grating, soft X-rays

## Abstract

We present here a newly developed software tool (called *PGMweb*) for computing and simulating the X-ray beam path through a plane grating monochromator (PGM), a key component in soft X-ray beamlines at modern synchrotron and free-electron laser facilities. A historical overview of the development of PGMs is presented, with special attention dedicated to the collimated PGM optical scheme found at several X-ray facilities worldwide. The analytical expressions that fully describe the geometry of a PGM are derived and have been implemented as functions in a Python library (*pyplanemono*). *PGMweb* is distributed as a web-based application that can be run in any modern browser without installation, making its use very straightforward for X-ray beamline designers and beamline scientists alike.

## Abstract 2

We present *PGMweb*, a visualization program for modelling the X-ray beam path as it is transmitted through plane grating monochromators.

## 1. Introduction

Monochromatic soft X-ray beams are indispensable for many areas of ongoing research. To perform X-ray absorption spectroscopy (XAS) at the *K*-edges of abundant main group elements (such as carbon and oxygen) as well as at the *L*-edges of the first row transition metals (such as iron or nickel), one needs to work in the soft X-ray range. Both XAS and X-ray photoelectron spectroscopy (XPS) performed with soft X-rays can shed light on the underlying physical and chemical processes at work in a variety of different research areas, from thin-film magnetism (Aich *et al.*, 2023 ▸) to catalytic processes (Venezia, 2003 ▸), and from strongly correlated electron systems (Gilmore *et al.*, 2021 ▸) to prototype battery materials (Liu *et al.*, 2019 ▸).

Achieving monochromation in the soft X-ray range is not straightforward. Below ∼2000 ▸ eV, one cannot use a double-crystal monochromator with Si(111) reflections, as the wavelength of the incoming photons is too large to satisfy the Bragg condition. In this energy range (∼50–2000 ▸ eV), potential crystal candidates with *d*-spacings large enough to accommodate the longer wavelengths are few and far between (Sutter, 2021 ▸). Moreover, the current capabilities in high-purity manufacturing of these exotic crystals are relatively limited, and in most cases their thermal properties are far from optimal, which is a significant factor considering the high heat load beams produced at large-scale X-ray facilities. These issues mean that double-crystal monochromators devoted to soft X-ray studies are rather impractical.

However, the dispersive property of reflective diffraction gratings (Ebert, 1889 ▸) offers the possibility of a monochromator design with adequate efficiency in the soft X-ray range. The pioneering work of Kunz *et al.* more than 50 years ago featured a monochromator which combines a plane mirror and plane grating (Kunz *et al.*, 1968 ▸), aptly named the plane grating monochromator (PGM). However, the mechanical design of the Kunz setup was rather complicated, as it required the mirror optic to be translated as well as rotated during operation. Later work performed at Zeiss during the 1980s (Riemer & Torge, 1983 ▸) showed that a single eccentric rotation of the mirror would, in effect, achieve the desired simultaneous translation and rotation. This mechanical arrangement was originally used at BESSY in the SX-700 type PGM design popular in the 1980s and 1990s (Petersen *et al.*, 1995 ▸) and is used in the vast majority of PGMs in operation today. Furthermore, PGMs are increasingly attractive monochromators for use in the notoriously difficult tender X-ray range (∼1500 eV to 4000 eV) due to recent advances in multilayer grating manufacturing (Wen *et al.*, 2024 ▸; Werner *et al.*, 2023 ▸).

**Figure 1:**
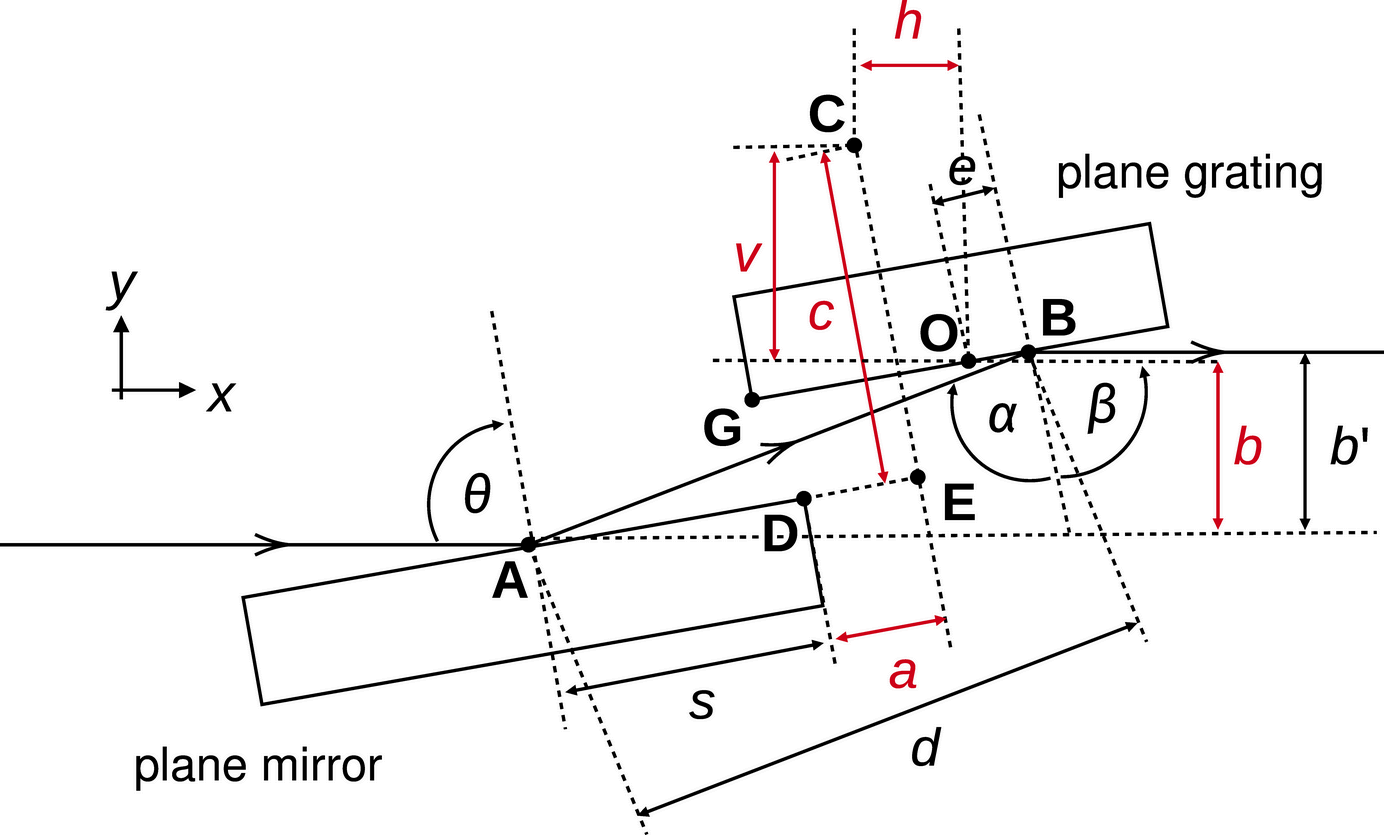
A schematic of a typical modern PGM geometry. Quantities highlighted in red are to be determined prior to the manufacturing of the PGM. All parameters presented in this figure are described in Table 1 ▸.

Diffraction from a grating is governed by the grating equation,
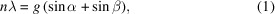
where *n* is the diffraction order and therefore takes integer values, λ is the selected wavelength of the radiation, *g* is the grating line density (normally defined in lines mm^−1^), and the angles α and β are as presented in Fig. 1 ▸ (see also Table 1 ▸). Throughout this work we follow the convention used in the X-ray data booklet (Thompson *et al.*, 2009 ▸), where α and β have opposite signs if they are on opposite sides of the normal. During operation, the mirror and the grating are rotated in a coordinated way to change α and/or β whilst ensuring that the outgoing rays remain parallel to the incoming rays. The grating disperses the X-rays in energy, and a small fraction of the dispersed radiation is then selected via a fixed downstream exit slit with a tunable opening in the dispersive direction. In this way a monochromatic beam is produced.

A common way to parameterize the relationship between α and β for a given grating geometry is through the property originally defined as the constant of fixed-focus (Petersen *et al.*, 1995 ▸), now commonly referred to as the grating *c*_ff_,
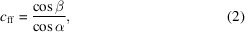
By substituting equation (2) into equation (1) and with suitable algebraic manipulations, one arrives at the updated grating equation commonly used today when operating PGMs,
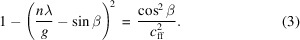
The only angular dependence of equation (3) is now in β, and equation (3) is essentially a quadratic equation in 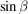. α can be calculated from β and a given *c*_ff_; and θ can be solved by imposing that the incoming and outgoing rays must remain parallel, that is, 2θ = α − β. Equation (3) calculates all three angles while only requiring the user of a PGM to dictate the desired diffraction order, the energy to be transmitted, and a value of *c*_ff_.

Nowadays a significant proportion of soft X-ray beamlines use the collimated PGM (cPGM) optical scheme proposed by Follath *et al.*, as it offers the ability to operate the PGM at different values of *c*_ff_ while maintaining a focused beam at the exit slit in the dispersive direction (Follath & Senf, 1997 ▸; Follath, 2001 ▸). This is in contrast to the older SX-700 scheme described by Petersen, where the beam was non-parallel in the vertical plane at the PGM, and it therefore required the use of the focusing properties of a plane grating (Petersen, 1982 ▸), and thus restricted the *c*_ff_ to a single value. Modifying *c*_ff_ in a cPGM beamline enables scientists to directly tune the resolving power, improve higher-order suppression, and/or customize the vertical divergence of the beam for a given experiment. The cPGM scheme continues to be one of the most popular soft X-ray beamline designs despite the fact that it has been more than two decades since its inception.

The mechanical design of a modern PGM is rather complicated, primarily stemming from the requirements that (1) the beam is always incident on the centre of the grating optical surface, and (2) the outgoing beam should remain parallel to the incoming beam. To achieve this, the mirror is rotated eccentrically, with the centre of rotation of the mirror located at **C** in Fig. 1 ▸ (Riemer & Torge, 1983 ▸). The point **C** is offset from the origin of the PGM coordinate system (**O**) by the vector 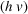 in the convention used here. The intercept of the incoming X-ray beam with the mirror surface is then a non-trivial function of the photon energy and the PGM *c*_ff_. While some time ago Pimpale *et al.* provided an analytical description of this geometrical design (Pimpale *et al.*, 1991 ▸), here we reintroduce some of the derivations described in that work but with an alternative parameterization that we feel is more practical for use by scientists at X-ray facilities.

**Figure 2:**
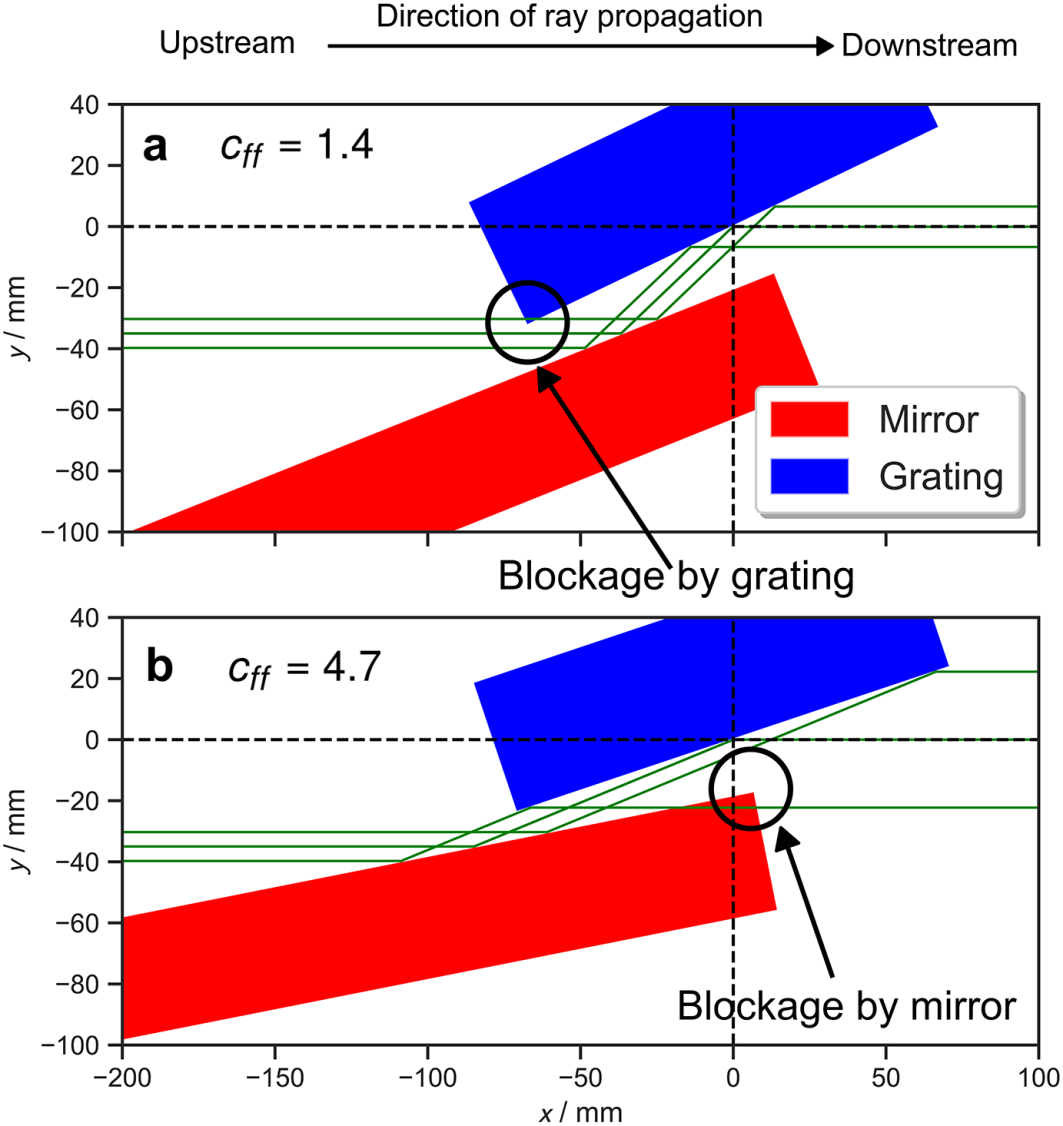
Two possible scenarios where self-blockage may occur. (*a*) Blocking by the upstream corner of the grating. (*b*) Blocking by the downstream corner of the mirror. Blockages are highlighted with circles.

**Figure 3:**
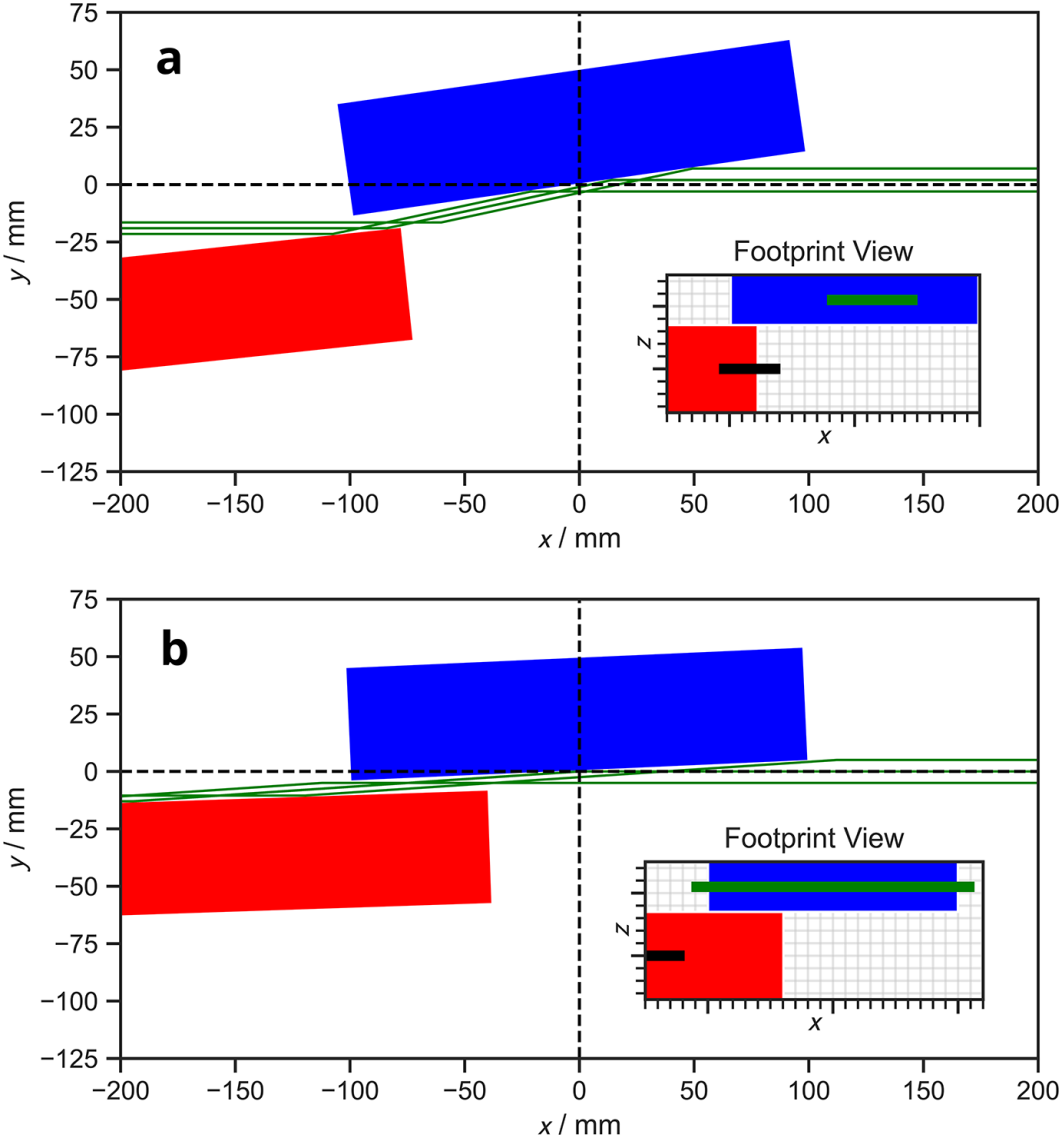
Two further possible scenarios where the beam is (*a*) only partially on the mirror surface, and (*b*) over-illuminating the grating. Two inset plots are provided for each scenario which gives the view of the top of the optical surfaces of the mirror and the grating as well as the footprints of the beam.

The mechanical evolution of the PGM as it performs an energy scan is potentially unintuitive. The complex nature of coupled rotations of the mirror and grating makes checking the feasibility of a set of PGM parameters non-trivial, as understanding the limits of operation for a PGM requires complete knowledge of its geometry. Limitations are encountered when:

(1) The beam is either blocked by the edges of the mirror or the grating leading to a partial/total loss of flux, Figs. 2 ▸(*a*) and 2 ▸(*b*).

(2) The beam is no longer incident on the optical surfaces of the mirror and/or the grating, Fig. 3 ▸(*a*).

(3) The grating or mirror is over-illuminated, Fig. 3 ▸(*b*).

In Fig. 3 ▸, we have included additional inset plots showing the ‘footprint view’ of the beam geometry within the PGM. The footprint view presents the beam footprints where the beam impinges on the respective optical surfaces, and does not show the propagating beam. It differs from a top view in two primary ways: (i) the grating has been offset horizontally for clarity so as not to overlap with the mirror, and (ii) the mirror and grating optical surfaces (and the corresponding beam footprints) are viewed normal to each optical surface. The horizontal direction is the direction of propagation of the rays (*x*) and the vertical direction is parallel to the widths of the optical elements (*z*). We have included the footprint view in *PGMweb*, as we feel that it is extremely useful both to beamline designers and beamline scientists.

In practice, a PGM is designed with a set of predetermined offsets which optimizes the performance of the PGM for the specific use case, *i.e.* energy range, or energy resolution. The optimized parameters that must be known prior to manufacturing are highlighted in red in Fig. 1 ▸. These parameters therefore define the range of energy and *c*_ff_ over which a given grating can transmit the whole beam for a particular PGM. We have therefore developed *PGMweb*, a software tool designed to help scientists to select these fixed parameters when designing a PGM. Our program also enables users to rapidly assess what is the lowest (or highest) energy that can be transmitted through a given PGM. Moreover, *PGMweb* provides an easy method for checking the transmission of an existing PGM with a new grating, which may have a different line density and therefore would operate in a different geometry (θ, α and β) compared with existing gratings.

## 2. The geometry of a PGM

In this section, we present the analytical expressions of all quantities of interest in a PGM. This includes the intercepts that the beam makes with both the grating and the mirror, as well as coordinates for the corners of both optics which have not been previously derived.

In all subsequent expressions, we define the horizontal as *x*, with positive *x* in the direction of ray propagation. The vertical is defined as *y*. The origin is defined to be at point **B** in Fig. 1 ▸, which is the location of the grating rotation axis. The bulk of the derivation will be performed in the (*x y*) plane. For simplicity, we present the derivations in two dimensions and introduce the *z* dimension at the end.

### 2.1. Intercepts with the mirror and the grating

Using quantities as they are defined in Fig. 1 ▸, the *x*–*y* coordinate of the intercept of the rays with the mirror is given by the expression
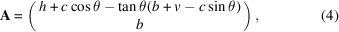
where *h*, *c*, *b* and *v* are all quantities known prior to the manufacturing of the PGM, and θ can be trivially calculated from the grating equation, equation (3).

Ideally the beam after the mirror would always be centred on the grating surface, so the grating is able to accept the largest beam possible at lower incident grazing angles (90° − α). In practice, the beam intercept (**B**) is slightly offset from the centre of the grating (**O**) by some finite distance *e* along the optical surface, as shown in Fig. 1 ▸. Using equation (4), an expression for *e* can be derived,
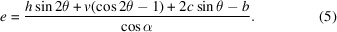


### 2.2. Minimization of *e*

To ensure that the beam hits the centre of the grating at a range of energies, the values of *b*, *c*, *v* and *h* must be carefully chosen. This can be done by minimizing the quantity *b*′ − *b*, the vertical displacement of the centre of the beam from the centre of the grating, which is equal to
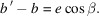
Written in full, from equation (5), 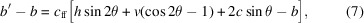
where we have substituted the definition of *c*_ff_. By satisfying the condition that the beam impinges on the centre of the grating, the left-hand side of the above equation is set to 0. After some rearrangement, this gives an expression for *b*,
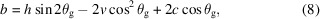
noting 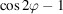 = 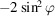 and that we have replaced θ with the grazing angle θ_g_ = π/2 − θ.

When θ_g_ is small (which is typical to ensure high reflectivities in the soft X-ray range), we can apply 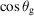 = 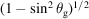 ≃ 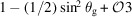. Substituting the approximation in equation (8) gives
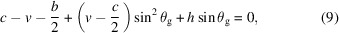
which would suggest that, in order to keep *b*′ − *b* small, the values of the offsets should be chosen such that *b* ≃ *c* ≃ 2*v* and *h* ≃ 0. This approximation has been applied to almost all PGMs at Diamond Light Source, *cf*. Table 2 ▸.

### 2.3. Corners of the grating

**Figure 4:**
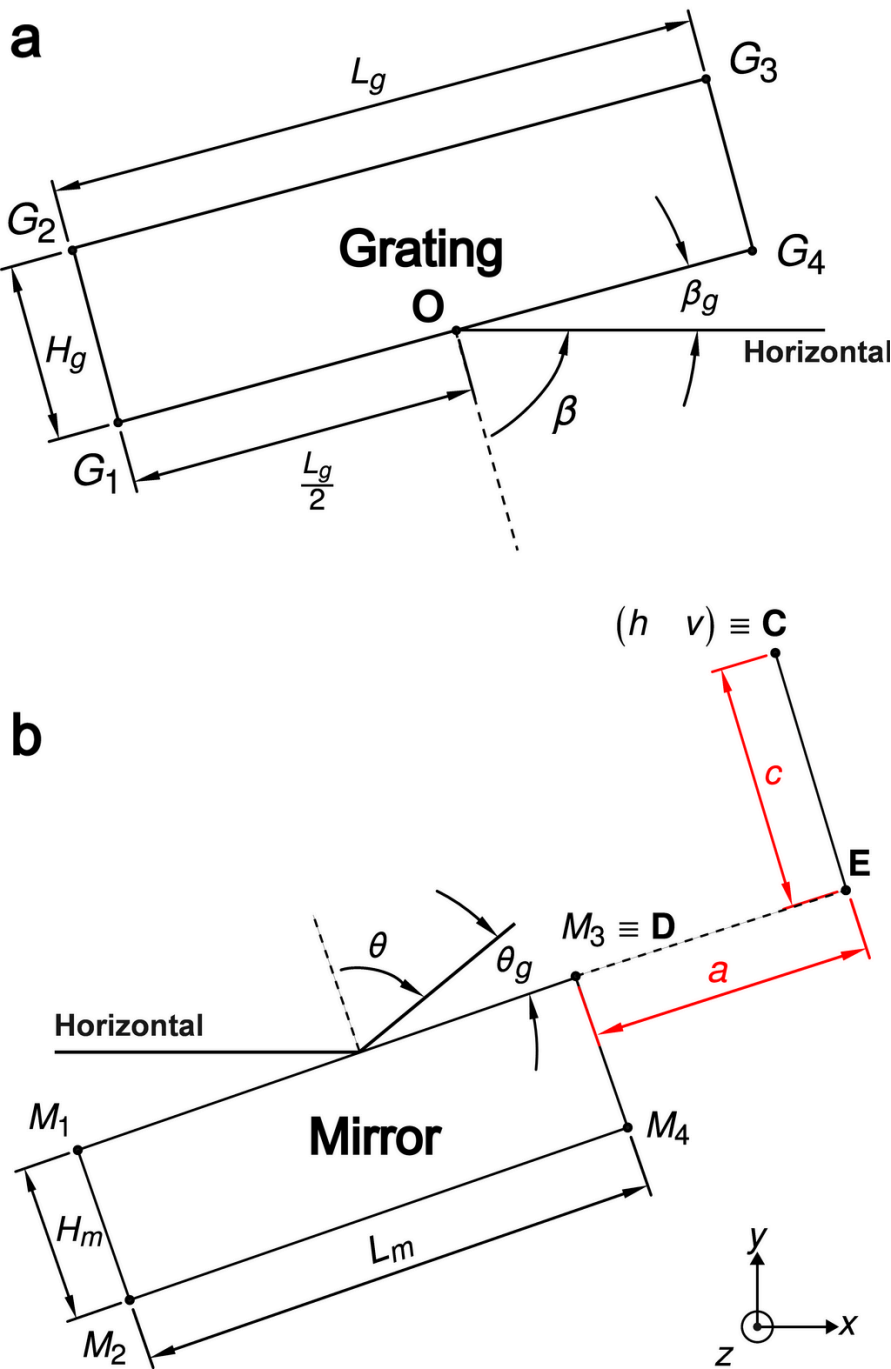
(*a*) A 2D side-view (*x*–*y*) projection of the grating optic, along with the dimensions of length (*L*_g_) and height (*H*_g_) denoted, and (*b*) an identical view of the mirror optic, with its dimensions *L*_m_ and *H*_m_ denoted.

Equally as important in the visualization of a PGM are the corners of the optics. The analytical expressions can be easily derived using trigonometry. Let the dimensions of the grating optic be defined as *L*_g_, *H*_g_ and *W*_g_, the length, height and width, respectively, where the length is the dimension in the tangential direction, the width is the dimension in the sagittal direction and height is the optical thickness. The corners of the grating are defined by the vectors *G*_1_, *G*_2_, *G*_3_ and *G*_4_ in the *x*–*y* plane; their expressions are 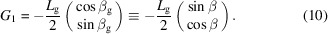
In Fig. 4 ▸(*a*), from the origin (**O**), the point *G*_1_ is in the fourth quadrant, where both the components *x* and *y* are negative. The expression for the coordinate is thus
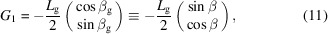

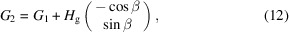

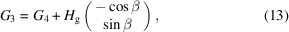

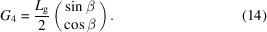
The third dimension *z* can be introduced trivially. A set of eight vertices of the grating can be found by placing the four points *G*_1_, *G*_2_, *G*_3_ and *G*_4_ in *z* = ±*W*_g_/2, so that the centre of the grating **O** is situated at the three-dimensional origin.

### 2.4. Corners of the mirror

In very much the same fashion, the vertices of the mirror can be found, with the added complication that the origin is in the middle of the grating optical surface. The mirror corners are given by the expressions
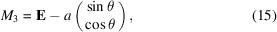

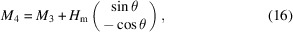

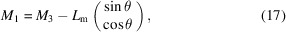

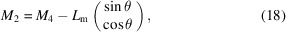
where the displacements are negative in all components.

The results derived from the expressions presented above were extensively compared with an alternative software tool developed at Diamond in Igor, which was described in a previous publication (Sutter *et al.*, 2023 ▸). This older software has been tested extensively during the design of several soft X-ray beamlines at Diamond, and the two software tools give results in complete agreement.

### 2.5. Beam size from an undulator source

Another area of consideration during PGM operation is the beam size from the source, more specifically, the height of the beam. For an undulator source at modern X-ray facilities, the vertical beam size at the PGM is significantly changing as a function of energy. Here we present the analytical expressions to calculate the beam height from a Gaussian undulator source (Peatman, 1997 ▸). The vertical root mean squared (RMS) photon source size (Σ) is given by
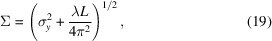
where σ_*y*_ is the vertical RMS electron beam size, λ is the wavelength of the beam and *L* is the length of the undulator. The vertical photon source divergence (Σ′) is given by a similar expression,
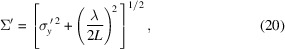
where 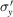 is the vertical electron beam divergence. It necessarily follows then that the final beam height at a plane distance *d* away is given by
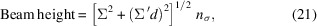
where *n*_σ_ is the number of standard deviations to include in considering the beam size.

## 3. Implementation

### 3.1. Python API

All analytical expressions that have been presented were compiled and coded into callable functions in Python. An object-oriented approach was employed to construct simulated instances of a mirror and a grating to form a PGM. This is distributed in the form of a Python library: *pyplanemono*. This code is freely available on Github (https://github.com/patrickwang27/pyplanemono) under an MIT licence, as well as on PyPI (https://pypi.org/project/pyplanemono/). The library additionally provides methods to draw PGM diagrams by performing simple ray tracing. The code computes all geometrical quantities of the PGM given a set of parameters defined using the class attributes of the PGM, mirror and grating. Notably, the package can also interface with *Matplotlib* and *Plotly* to produce diagrams of the PGM. We have therefore included animations of a PGM performing both an energy scan as well as a *c*_ff_ scan in the supporting information.

### 3.2. PGMweb

**Figure 5:**
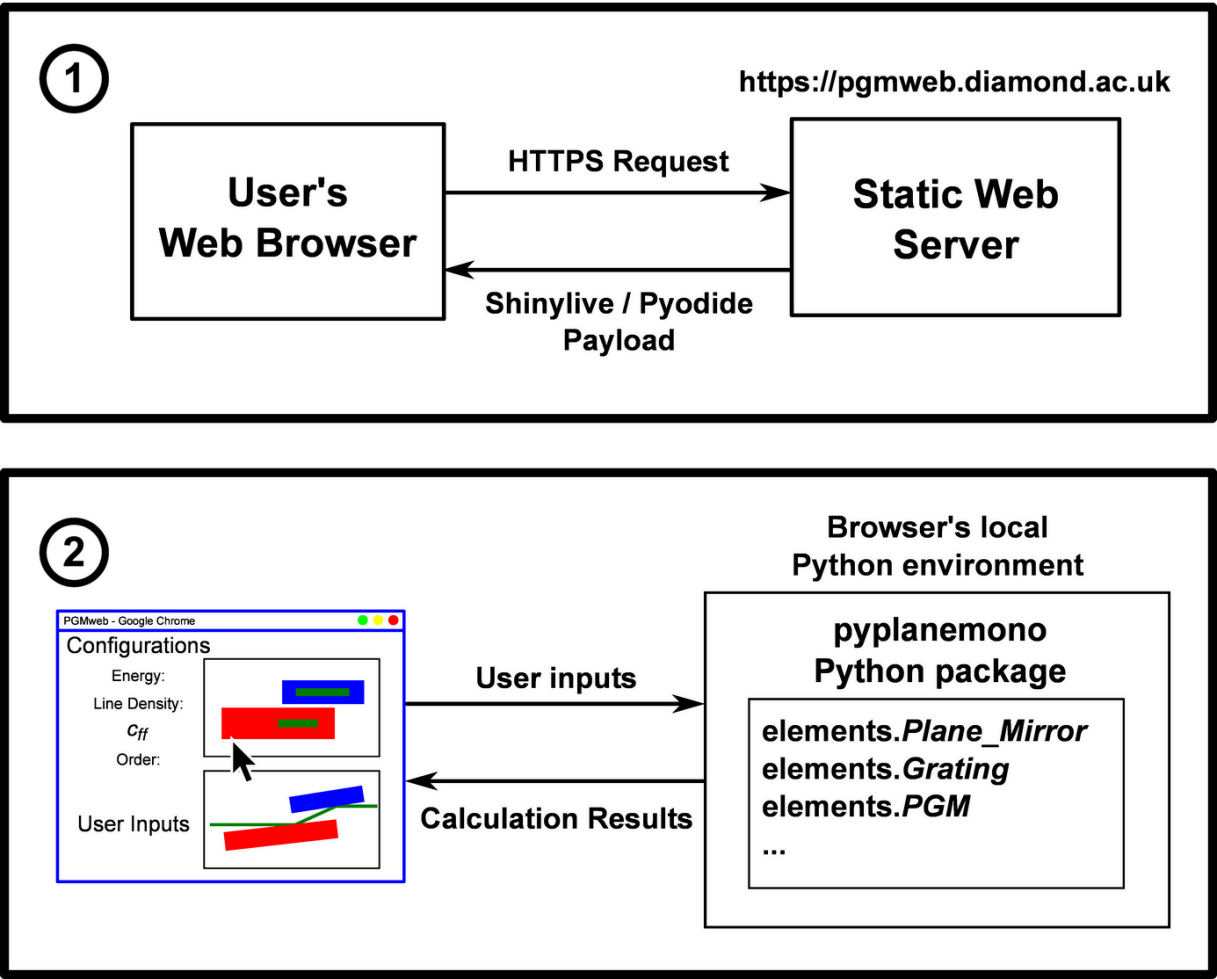
A cartoon depiction of using *PGMweb* to illustrate the role each entity serves.

**Figure 6:**
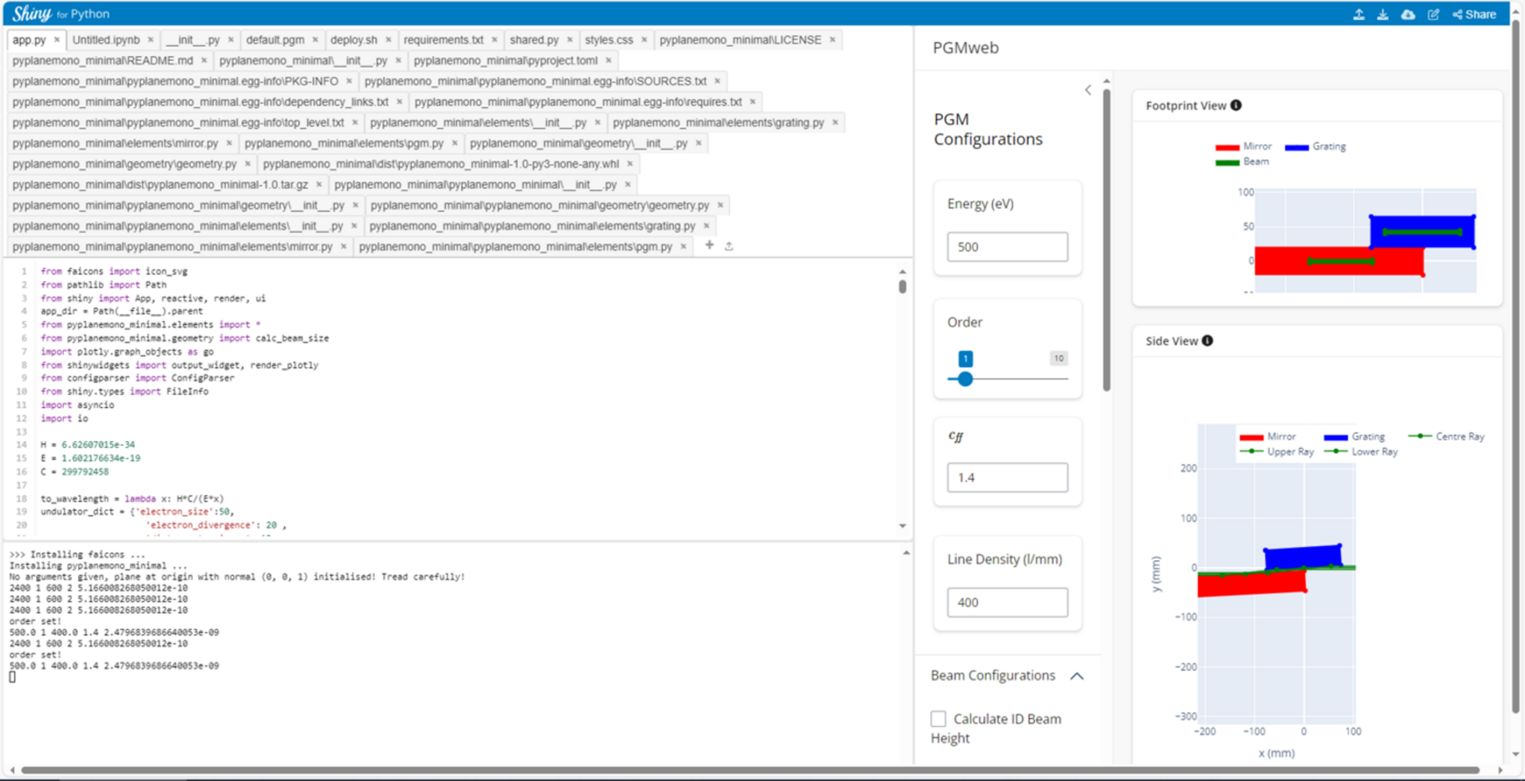
A screenshot of the *PGMweb* app in the edit mode. This mode is accessed by visiting https://pgmweb.diamond.ac.uk/app/edit/.

*PGMweb* (https://pgmweb.diamond.ac.uk) is an extension of the underlying calculation software package. A front-end graphical user interface (GUI) was developed using Shiny for Python (https://shiny.posit.co/py/), a dashboarding library commonly used in data science. This provides a GUI that can run in any modern browser that supports JavaScript. To simplify the user experience and avoid manual installations, we have opted to distribute the software as an online application with Shinylive (https://github.com/posit-dev/shinylive). Shinylive bundles the GUI code with a minimal Python environment in WebAssembly [Pyodide (https://pyodide.org/en/stable/)] to produce files for a static website. The workflow presented in Fig. 5 ▸ shows that upon visiting the *PGMweb* website the server returns a set of files containing everything necessary to run a minimal Python environment within the browser. After the user’s local setup is complete (typically within 1 min), all calculations are run by the *pyplanemono* package within the browser, at which point the server ceases to play any role. The delay experienced by the user would have been considerably less had the calculation been performed on a separate remote Python server. Shiny also includes a feature which makes any code executed completely transparent to the user and can be modified on-the-fly to better suit the user’s needs. This can be done by accessing the edit function of Shiny, as shown in Fig. 6 ▸.

### 3.3. Features

**Figure 7:**
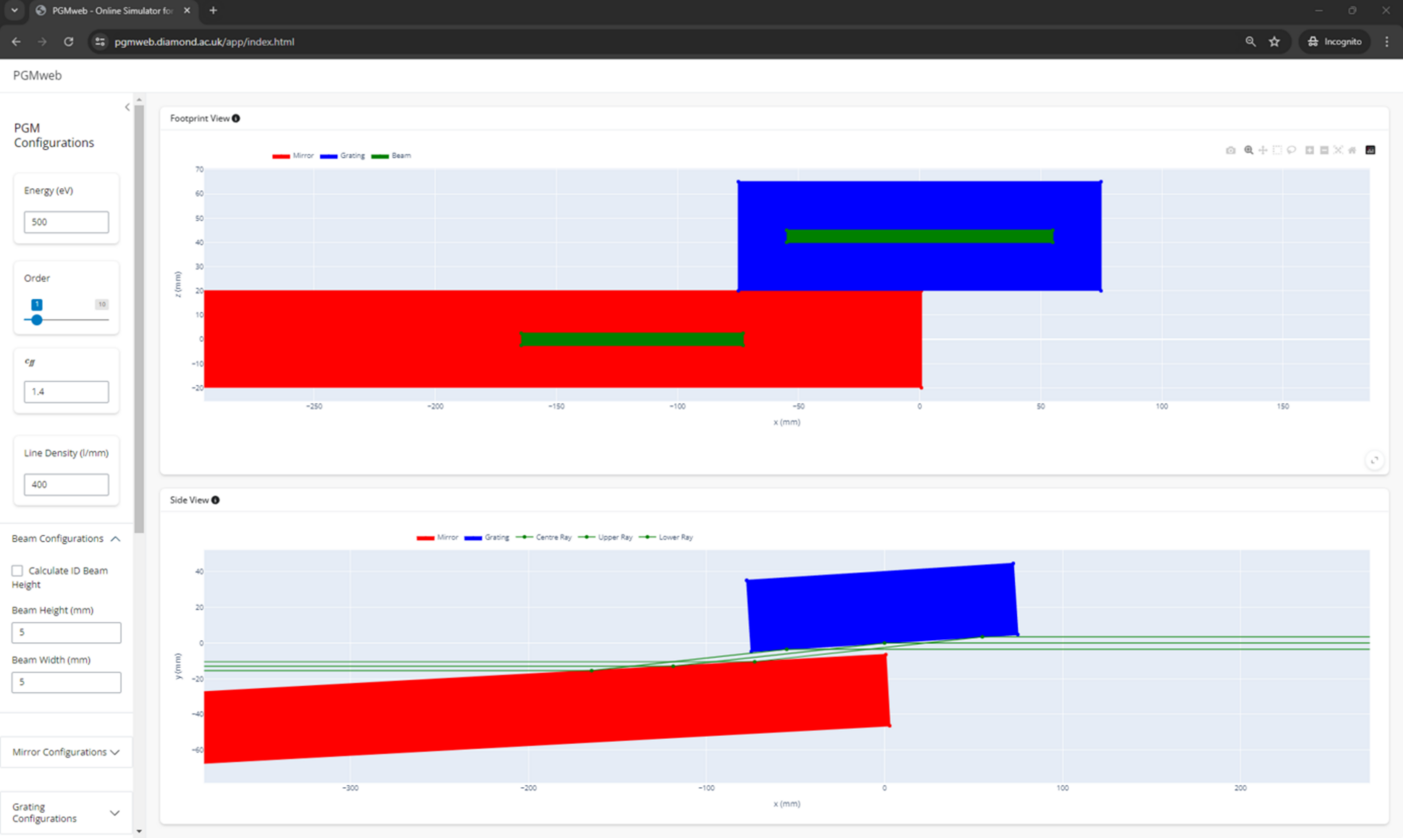
A screenshot of the *PGMweb* interface in a Google Chrome browser.

The interface allows the user to define all key parameters that dictate the geometry of a PGM, Fig. 7 ▸. The energy, *c*_ff_, the grating line density and the diffraction order are highlighted as four primary controls and occupy a prominent space in the GUI to allow quick and easy reading/changing of their values. Controls for the finer details are included as well; the user can fully specify the geometry of a PGM using the appropriate offsets previously defined. The software also has the ability to compute the offset parameters *h*, *c* and *v* when the user specifies the offset *b*, using the approximation described in Section 2.2. The software computes the geometry of the PGM and shows a plot of the beam footprints on the optical surfaces, as well as a plot of the side-view diagram of the rays propagating through the PGM. This can be very helpful in quickly determining whether or not the whole beam can be transmitted through a given PGM geometry. We note the assumption that the size of the clear aperture of the optics is equivalent to the actual physical size of the components. The beam dimensions can be specified manually or computed by the programme from user-specified parameters for an undulator source (*cf*. Section 2.5).

The figures can be downloaded as vector graphic files, and the application also allows the user to export the PGM configuration to a human-readable TOML file which can be saved locally and imported at a later time if required.

## 4. Conclusion

In this paper, we have derived expressions for various geometrical parameters which allow the geometry of a PGM to be computed in its entirety. The expressions are implemented as class methods in a Python package called*pyplanemono*, which provides a set of robust application programming interfaces (APIs). The graphical front-end provides a quick and accessible tool for checking the geometry of a PGM given a set of parameters. We highlight that *PGMweb* does not itself identify if any part of the beam will not be transmitted due to the geometry of the PGM, but presents visualizations which make any blockages apparent to the user (see for example Figs. 2 ▸ and 3 ▸).

We note that the expressions used in *PGMweb* assume that the condition 2θ = α − β always holds, as is intended in most real applications. Any misalignment of a beamline that would introduce an angular offset will lead to a more complicated analytical expression for the grating equation than that presented in equation (3), and could also affect the transmission of the PGM for a given energy and *c*_ff_.

The development of the underlying Python library described here opens up the possibility for interfacing it with ray-tracing software such as *SHADOW3* in the future (Sanchez del Rio *et al.*, 2011 ▸), where the blockages could be accounted for natively in ray-tracing simulations of soft X-ray beamlines. Such ray-tracing simulations could be scripted to be performed over a wide range of diffraction orders, energies and values of *c*_ff_, providing a full picture of the parameter space accessible with existing or planned cPGM beamlines. We plan to explore these possibilities in our future work.

## Supplementary Material

Animations of a plane grating monochromator performing a cff scan. DOI: 10.1107/S1600577524011603/tv5072sup1.gif

Animation of a plane grating monochromator performing an energy scan. DOI: 10.1107/S1600577524011603/tv5072sup2.gif

## Figures and Tables

**Table 1:** The complete list of parameters that define the geometry of a PGM and the commonly assigned variables

Description	Parameter name	Fixed or varying in operation
Displacement vector of the centre of beam footprint on the plane mirror	**A**	Varying
Displacement vector of the plane grating rotation axis (origin of the *x*, *y* coordinate system)	**O**	Fixed
Displacement vector of the centre of beam footprint on the plane grating	**B**	Varying
Displacement vector of the plane mirror rotation axis	**C**	Fixed
Displacement vector of downstream edge of the plane mirror	**D**	Varying
Projection of **C** onto plane mirror surface	**E**	Varying
Displacement vector of the bottom left corner of the grating	**G**	Varying
Distance between **D** and E	*a*	Fixed
Vertical displacement between **B** and incident beam	*b*	Fixed
Vertical displacement between exit beam and incident beam	*b*′	Varying
Rotation radius for plane mirror	*c*	Fixed
Distance between **A** and **B**	*d*	Varying
Distance between **O** and **B**	*e*	Varying
Vertical displacement between **C** and **O**	*v*	Fixed
Horizontal displacement between **C** and **O**	*h*	Fixed
Distance between **A** and **D**	*s*	Varying
Normal angle of incidence on the mirror	θ	Varying
Normal angle of incidence on the grating	α	Varying
Normal angle of diffraction from the grating	β	Varying

**Table 2:** A compendium of all PGMs installed at the Diamond Light Source and their relevant offsets

Beamline	Short summary	*a* (mm)	*b* (mm)	*v* (mm)	*c* (mm)	*h* (µm)
I05 (Hoesch *et al.*, 2017 ▸)	Angle-resolved photoelectron spectroscopy	5	35	19.37	36.88	−190.36
I06 (Dhesi *et al.*, 2010 ▸)	Photoelectron emission microscopy	40	15	7.67	15.25	0
I08	Scanning X-ray microscope	35	10	5.054	10.054	0
I09 (Lee & Duncan, 2018 ▸)	Surface and interface structural analysis	50	16	8.111	16.111	−4
I10	Advanced dichroism experiments	40	15	7.67	15.25	0
I21 (Zhou *et al.*, 2022 ▸)	Resonant inelastic X-ray scattering	40	15	7.5	15	0
B07c (Held *et al.*, 2020 ▸)	Versatile soft X-ray (VerSoX) beamline	40	13	6.5	13	0
B07b (Grinter *et al.*, 2024 ▸)	Versatile soft X-ray (VerSoX) beamline	40	24	12	24	0
B24 (Harkiolaki *et al.*, 2018 ▸)	Full-field cryo-X-ray microscopy for life sciences	50	10	5	10	0
