# Supplementary figures and images for: PGMweb: an online tool for visualizing the X-ray beam path through plane grating monochromators
Source: J Synchrotron Radiat. 2025 Jan 1;32(Pt 1):261–8. doi: 10.1107/S1600577524011603 (PMC11708866; doi:10.1107/S1600577524011603)

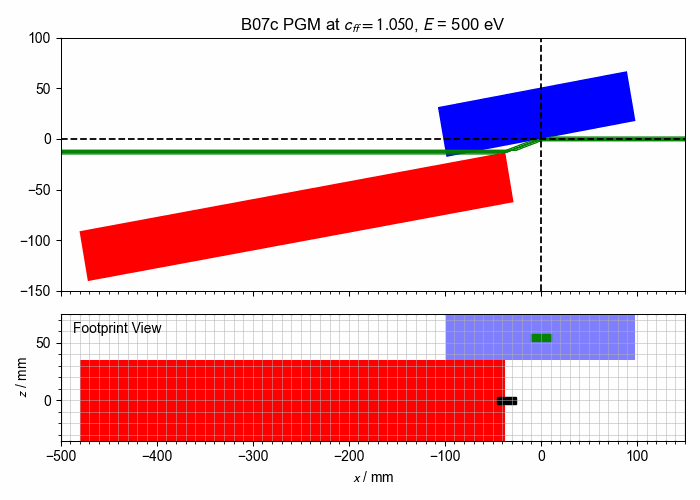

Supplement: Supplementary file 1 [file s-32-00261-sup1.gif]

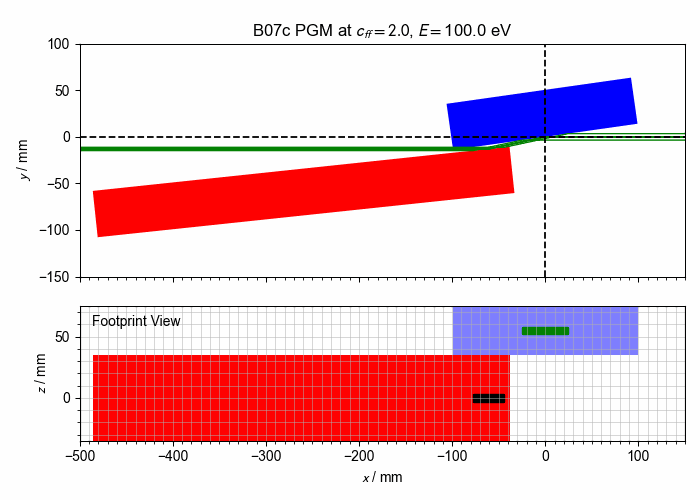

Supplement: Supplementary file 2 [file s-32-00261-sup2.gif]
